# Infantile Homocystinuric Urolithiasis With Staghorn Calculus: A Case Report of Nephron-Sparing Surgical Salvage in an Intraparenchymal Renal Pelvis

**DOI:** 10.7759/cureus.111751

**Published:** 2026-06-29

**Authors:** Jevina Daphne Winnifred, Jayatheertha Joshi, Kishan B Shetty, Leo F Tauro

**Affiliations:** 1 General Surgery, Father Muller Medical College Hospital, Mangalore, IND; 2 Pediatric Surgery, Father Muller Medical College Hospital, Mangalore, IND

**Keywords:** homocystinuria, infantile urolithiasis, intraparenchymal pelvis, open pyelolithotomy, staghorn calculus

## Abstract

Pediatric urolithiasis, though uncommon, carries significant morbidity when driven by underlying metabolic disorders. Classical homocystinuria, caused by cystathionine beta-synthase (CBS) deficiency, is a rare autosomal recessive inborn error of sulfur amino acid metabolism. While ectopia lentis, skeletal anomalies, and thromboembolic events define its classical triad, urolithiasis presenting within the first year of life remains exceptionally rare in surgical literature. A one-year-old male, born to consanguineous parents with known homocystinuria, presented with gravel-like urinary deposits since five months and progressive dysuria since six months. Ultrasonography revealed a 10 mm right renal calculus, a 23 mm vesical calculus, and features of chronic cystitis. He underwent cystolithoscopy, open cystolitholapaxy, and double-J (DJ) stenting. Repeat imaging at two months revealed a 17 mm right staghorn calculus with mild hydronephroureterosis. Intraoperatively, an intraparenchymal pelvis was encountered with the staghorn calculus densely adherent to the indwelling stent, precluding conventional retrieval. Nephrectomy was deferred in favour of nephron-sparing surgery. The calculus was extracted en bloc, a fresh DJ stent inserted, and watertight ureteral re-anastomosis performed. Homocystinuria can drive aggressive multi-level urolithiasis in infancy. Open pyelolithotomy with ureteral re-anastomosis remains a viable nephron-saving strategy when minimally invasive approaches are anatomically unfeasible. Sustained metabolic-surgical multidisciplinary management is essential for long-term renal preservation.

## Introduction

Urolithiasis in the paediatric population, though infrequent, represents a clinically challenging entity with a rising incidence over the past two decades. Childhood urolithiasis has evolved from a predominantly infectious aetiology to a predominantly metabolic one, necessitating a comprehensive biochemical evaluation in all affected children [1]. A metabolic cause is identifiable in 40-50% of cases, and early recognition is indispensable to prevent progression to chronic renal failure [2].

Classical homocystinuria, caused by cystathionine beta-synthase (CBS) deficiency, is a rare autosomal recessive disorder of sulfur amino acid metabolism with a worldwide prevalence of 1:1,800 to 1:900,000 [3,4], classically manifesting as a tetrad of ocular (ectopia lentis), skeletal (marfanoid habitus), vascular (thromboembolism), and neurological (intellectual disability) features [5]. Urolithiasis falls outside this recognised tetrad, and its occurrence as a presenting feature in infancy remains exceptionally rare and largely unreported in urological and surgical literature.

Staghorn calculi in children under four years of age are themselves uncommon, with metabolic aetiology and recurrent urinary tract infection identified as the primary contributory factors [6]. The surgical management of staghorn calculi in the paediatric age group is further complicated by the small calibre of the collecting system, limited surgical access, and the high risk of renal compromise. We report this unique case to highlight homocystinuria as an underrecognised cause of aggressive infantile urolithiasis, and to describe a novel intraoperative salvage strategy in the setting of an intraparenchymal renal pelvis.

To the best of our knowledge, this represents the first documented report of homocystinuria presenting as staghorn urolithiasis in infancy, managed by nephron-sparing open pyelolithotomy in the anatomically challenging setting of an intraparenchymal renal pelvis; this case is reported to contribute to the limited literature on metabolic urolithiasis in early infancy and to guide surgical decision-making in similarly complex scenarios.

## Case presentation

In September 2025, a one-year-old male child, born of a consanguineous union and a known case of homocystinuria diagnosed on newborn metabolic screening, was brought to the outpatient department by his mother with a history of passage of gravel-like material in urine since the age of five months. The mother also reported increased urinary frequency and dysuria since six months of age. There was no history of fever, gross haematuria, or failure to thrive. On examination, the child was haemodynamically stable with normal anthropometric parameters for age. Abdominal examination revealed no palpable renal mass. Urinalysis demonstrated pyuria and crystalluria. Ultrasonography of the abdomen and pelvis revealed a 10 mm right renal calculus without hydronephrosis, a 23 mm calculus within the urinary bladder, and bladder wall thickening with mucosal irregularities consistent with chronic cystitis (Figure 1). Serum renal function tests were within normal limits. After appropriate pre-operative optimisation, the child underwent cystolithoscopy, open cystolitholapaxy, and double-J (DJ) stenting under general anaesthesia. The postoperative course was uneventful.

**Figure 1:**
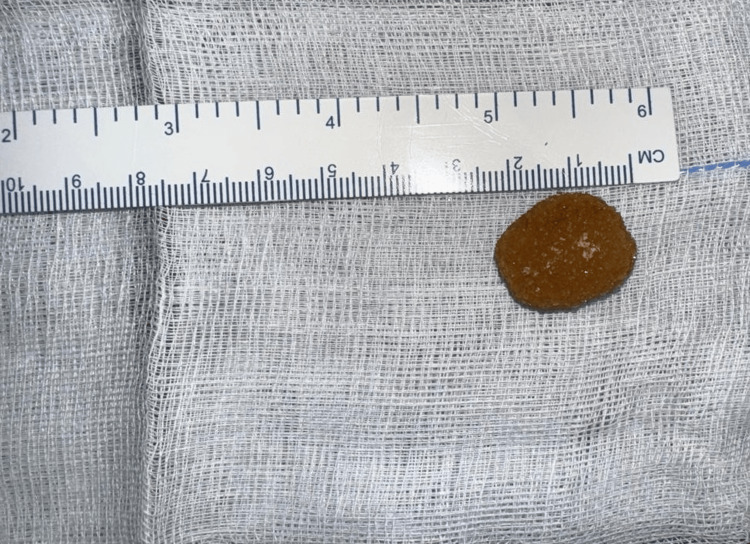
Gross photograph of the vesical calculus retrieved by open cystolitholapaxy. The stone measures approximately 17–20 mm in greatest dimension and demonstrates a granular, lobulated surface with deep amber-brown colouration, morphologically consistent with a mixed metabolic-infective calculus in the setting of homocystinuria and concurrent urinary tract infection.

At the two-month follow-up, the child presented with recurrence of dysuria and passage of gravel. Repeat ultrasonography demonstrated a 17 mm echogenic calculus with posterior acoustic shadowing in the right kidney with mild hydronephroureterosis (HUN), and non-contrast computed tomography (NCCT) of the abdomen and pelvis confirmed a hyperdense staghorn calculus measuring 6x14x16 mm in the right renal pelvis with an attenuation of 767 HU. (Figure 2). In view of the progressive stone burden and the presence of the indwelling DJ stent, a decision was made to proceed with open right pyelolithotomy.

**Figure 2:**
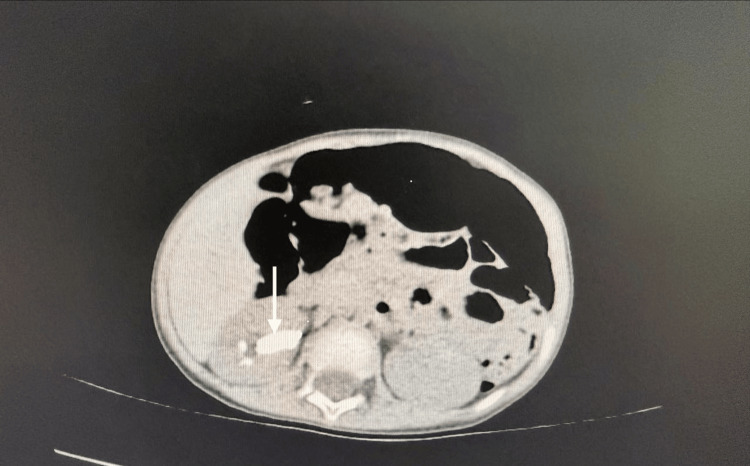
Axial view CT abdomen. Demonstrated dilated bowel loops with displacement by the right renal mass and evidence of staghorn nephrolithiasis.

Intraoperatively, the renal pelvis was found to be of the intraparenchymal variety - entirely embedded within the renal parenchyma - providing severely limited surgical access. The staghorn calculus was found to be densely adherent to and encrusted around the existing DJ stent, making conventional retrieval through a standard pyelotomy incision anatomically impossible. The possibility of nephrectomy was considered, given the technical complexity; however, in view of the child's age and the goal of renal preservation, an aggressive nephron-sparing approach was pursued.

The ureter was carefully transected at the pelvi-ureteric junction. Through this wider access, the staghorn calculus was successfully extracted en bloc along with the encrusted DJ stent (Figure 3). Saline irrigation was performed to ensure complete clearance of all residual fragments. A fresh DJ stent was then inserted through the renal pelvis into the ureter. The ureter was re-anastomosed to the intraparenchymal pelvis with interrupted 4-0 Vicryl sutures in a meticulous watertight fashion. A closed-suction drain was secured in the retroperitoneum near the renal pelvis. The drain output was minimal, and it was removed on postoperative day five. The child was discharged on postoperative day seven in a stable condition with the DJ stent in situ.

**Figure 3:**
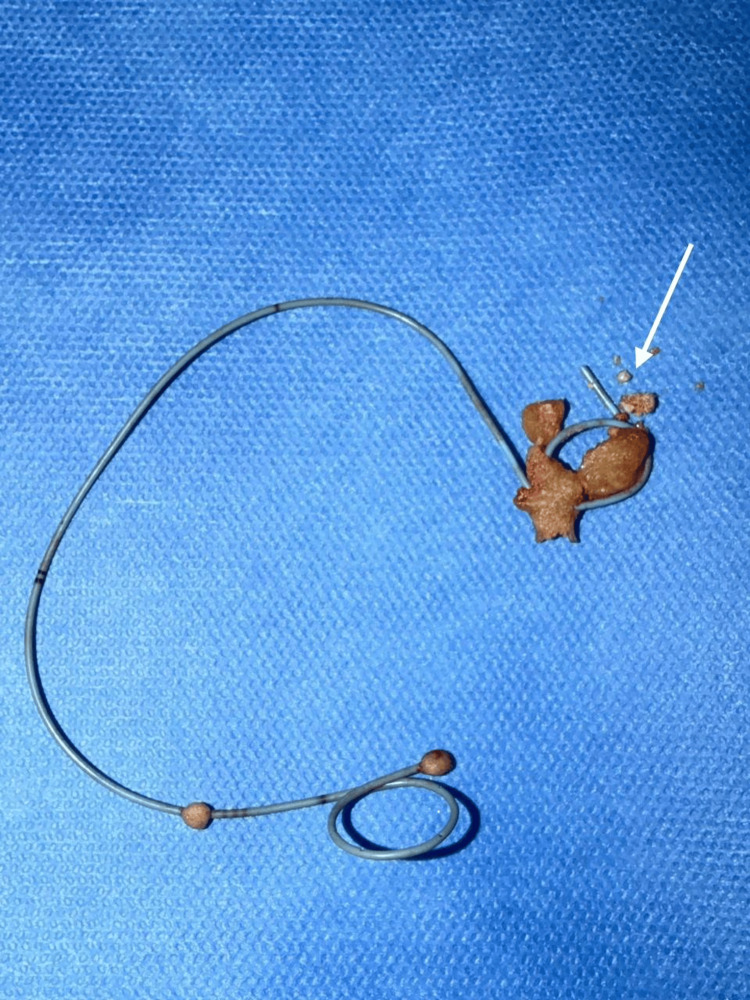
En bloc extraction specimen. Demostrated the double-J ureteric stent with densely adherent staghorn calculus fragments at the proximal (renal) end. The upper pigtail curl is completely encased within fragmented amber-brown calculous material, confirming the intraoperative finding of stent-stone fusion that precluded conventional retrieval.

At the last follow-up, nine weeks post-operatively, the patient remains clinically well with a serum creatinine of 0.9 mg/dl and no features of obstructive uropathy. Renal ultrasonogram demonstrated the Right DJ stent in situ with normal corticomedullary differentiation with echogenic foci measuring 5 mm with posterior acoustic shadowing noted in the lower pole and renal pelvis, suggestive of residual/recurrent calculi. A plan for elective DJ stent removal and further urological evaluation was made at this visit. The patient has been referred to the metabolic team for optimisation of homocysteine control and dietary compliance, with a structured surveillance protocol instituted for renal monitoring every six months.

## Discussion

This case represents one of the earliest documented instances of homocystinuria presenting as multi-level urolithiasis within the first year of life. Skovby et al. in their revisit to the natural history of CBS deficiency, emphasised that the classic manifestations - ectopia lentis, skeletal deformities, and thromboembolism - typically emerge in the first or second decade of life [7]. Urolithiasis is conspicuously absent from that natural history, making its occurrence in a five-month-old infant a clinically singular finding. Morris et al. noted in the international CBS deficiency guidelines that homocysteine accumulation promotes vascular and connective tissue damage through oxidative stress; by extension, analogous nephrotoxic effects on the tubular epithelium and disruption of cystathionine-mediated crystal inhibition may underpin stone formation in this disorder, though this mechanism warrants direct investigation [8].

The index case demonstrated two distinct and rapidly successive phases of stone disease: vesical lithiasis at presentation requiring cystolitholapaxy, followed within two months by staghorn nephrolithiasis - an exceptionally aggressive trajectory even by the standards of inherited metabolic stone diseases. Sas et al. documented that children with metabolic urolithiasis carry a substantially elevated lifetime risk of stone recurrence compared to adults, particularly when the underlying metabolic defect remains uncorrected [1]. Poor compliance with betaine supplementation and dietary methionine restriction in the index patient likely abrogated the only effective brake on stone progression. In comparison, Sharma et al. reported a 20-month-old with cystinuria - another autosomal recessive sulphur amino acid disorder - who presented with staghorn calculi; like our patient, the stone disease was bilateral and demonstrated rapid recurrence [9]. The parallel between cystinuria and homocystinuria in driving early-onset, high-burden urolithiasis points to a broader category of 'sulphur aminoaciduric urolithiasis' that may benefit from a unified metabolic surveillance protocol in paediatric urology. The ultrasonographic finding of chronic cystitis with bladder wall thickening and mucosal irregularities in this infant introduced a second, independent driver of stone growth. De Castro et al., in their paediatric case series of staghorn calculi, identified urinary tract infection as the dominant aetiological cofactor in children under four years, with metabolic disease as a close second [10]. The two factors are synergistic rather than additive: recurrent urinary tract infection with urease-producing organisms such as Proteus mirabilis hydrolyses urinary urea into ammonia, raising urinary pH and promoting supersaturation of magnesium ammonium phosphate (struvite). Stone analysis in this case revealed a mixed composition of cystine, calcium oxalate, and struvite. The cystine component is consistent with the underlying sulfur amino acid metabolic defect, as homocystine and cystine share renal tubular transport pathways, and reduced cystine solubility at urinary pH below 7.0 promotes crystallisation [3,4]. The calcium oxalate fraction likely reflects hyperoxaluria secondary to impaired transsulfuration, a well-recognised metabolic consequence of CBS deficiency [8]. The struvite component reflects a superimposed urinary tract infection, which promoted urease-mediated alkalinisation and struvite precipitation. This polymicrobial lithogenic milieu - metabolic, infective, and obstructive - explains both the aggressive growth pattern observed in our patient (10 mm to staghorn within five months) and the encrustation of the indwelling DJ stent. Mixed-composition stones of this nature are particularly refractory to lithotripsy and typically mandate open surgical extraction, as was necessary in the present case.

The pivotal intraoperative challenge was the intraparenchymal renal pelvis - a variant in which the pelvis is entirely encased within the renal sinus parenchyma rather than projecting extrarenally. Vijayakumar et al., reporting their robotic extended pyelolithotomy series for partial staghorn calculi, explicitly emphasised that pre-operative characterisation of the degree of intrarenal versus extrarenal pelvic component is the single most critical determinant of both surgical approach and operative complexity [11]. In their series, cases with a predominantly intrarenal pelvis required a significantly longer operative time and a modified V-shaped pyelotomy incision to achieve adequate stone access. In the present case, this anatomical detail was not fully anticipated pre-operatively, underscoring a critical lesson: CT urography with three-dimensional pelvicalyceal reconstruction should be mandatory before any open or minimally invasive pyelolithotomy in the paediatric age group, particularly when prior stent placement and a metabolic stone diathesis predict a hostile surgical field.

Percutaneous nephrolithotomy (PCNL) is the guideline-recommended first-line modality for large paediatric renal stone burdens. Desai et al. reported an overall stone-free rate of 79% with PCNL in paediatric urolithiasis, with staghorn stones carrying a 34% ancillary procedure requirement [12]. However, PCNL feasibility is contingent on an adequately dilated collecting system, the absence of dense perinephric adhesions, and - critically - the absence of a calculus-stent complex. In our case, all three conditions were unfavourable. The staghorn calculus had fully encrusted the indwelling DJ stent, a complication catalogued by Thapa et al., who described full-thickness calcification around retained stent biomaterial as a well-documented consequence of metabolic stone diathesis and suboptimal urinary drainage [13]. Thapa et al.'s case, like ours, ultimately required open surgical extraction with watertight pelvic closure - and similarly achieved complete stone clearance without nephrectomy [13]. The decision to transect the ureter to create wider access for en bloc extraction of the calculus-stent complex, followed by primary re-anastomosis, is a departure from standard pyelolithotomy technique but was validated by a complication-free recovery and drain removal on postoperative day five.

The long-term surgical outcome in CBS deficiency is inextricably linked to metabolic control. Morris et al. recommend a target total plasma homocysteine of less than 50 µmol/L [7]. This requires a multi-pronged approach: (1) a low-methionine diet with methionine -restricted amino acid supplements; (2) betaine supplementation (100-250 mg/kg/day) to enhance homocysteine remethylation; and (3) pyridoxine (vitamin B6) in responsive phenotypes [8]. Mudd et al. demonstrated in their landmark natural history cohort that treated patients had dramatically lower rates of thromboembolic and end-organ complications compared to untreated counterparts [3]. The recurrence of stone disease in our patient within two months of the initial surgery, in the context of documented non-compliance with both diet and betaine, directly corroborates the critical role of metabolic control. Stone-free status achieved surgically is therefore only a reprieve unless underpinned by sustained biochemical remission; Hence, we recommend that all children with homocystinuria and urolithiasis be co-managed with a metabolic team, with routine monitoring of plasma homocysteine, urinary pH, and renal ultrasound every six to 12 months.

This case underscores the importance of a joint surgical-metabolic approach in paediatric homocystinuric urolithiasis. Surgical intervention addresses the immediate obstructive and infective burden, but durable stone-free status is contingent on sustained metabolic control. Failure to achieve target homocysteine levels will predictably result in recurrent stone disease, progressive renal injury, and repeated operative morbidity

## Conclusions

This case underscores that homocystinuria, though classically characterised by ocular and skeletal manifestations, can present as rapidly progressive and surgically complex urolithiasis within the first year of life. The combination of metabolic stone diathesis, superimposed urinary infection, intraparenchymal renal pelvis, and stent-calculus encrustation created a formidable intraoperative challenge that is not anticipated by standard surgical algorithms. Open pyelolithotomy with ureteral transection, en bloc calculus-stent extraction, and watertight re-anastomosis proved to be an effective nephron-sparing salvage strategy. This case highlights the indispensable role of pre-operative CT pelvicalyceal mapping, early metabolic diagnosis, rigorous dietary compliance, and sustained multidisciplinary follow-up in achieving durable renal preservation in this rare cohort of patients.
